# Testing the feasibility and safety of feeding preterm infants fresh mother’s own milk in the NICU: A pilot study

**DOI:** 10.1038/s41598-018-37111-7

**Published:** 2019-01-30

**Authors:** Huiqing Sun, Shuping Han, Rui Cheng, Mingyan Hei, Foteini Kakulas, Shoo K. Lee

**Affiliations:** 1 Department of Neonatology, Children’s Hospital of Zhengzhou University, Zhengzhou, China; 2 Department of Neonatology, Henan Children’s Hospital, Zhengzhou, China; 3 Department of Neonatology, Zhengzhou Children’s Hospital, Zhengzhou, China; 4 Department of Pediatrics, The Affiliated Obstetrics and Gynecology Hospital of Nanjing Medical University, Nanjing Maternity and Child Health Care Hospital, Nanjing, China; 5 Department of Neonatology, Children’s Hospital of Nanjing Medical University, Nanjing, China; 6 Department of Pediatrics, The Third Xiangya Hospital of Central South University, Changsha, China; 7 Neonatal Center, Beijing Children’s Hospital, Capital Medical University, Beijing, China; 8 Medical School, Faculty of Health and Medical Sciences, The University of Western Australia, Crawley, Western Australia Australia; 9 Maternal-Infant Care Research Centre, Mount Sinai Hospital, Toronto, Ontario, Canada; 10 Department of Paediatrics, Department of Obstetrics & Gynecology, and Dalla Lana School of Public Health, University of Toronto, Toronto, Ontario, Canada; 11 Department of Paediatrics, Sinai Health System, Toronto, Ontario, Canada

## Abstract

Necrotizing enterocolitis (NEC) is the leading cause of death among infants born at <30 weeks’ gestation, but donor human milk can reduce the incidence of NEC. Unfortunately, freezing or pasteurizing human milk deactivates beneficial bioactive components. We evaluated the feasibility, safety, and impact of feeding very preterm infants fresh (unprocessed) mother’s own milk within 4 hours of expression. In our multicentre prospective cohort analytic study, we fed 109 control and 98 intervention infants previously frozen donor or mother’s own milk; only the intervention group was fed fresh mother’s own milk once daily from enrollment until 32 weeks’ corrected age. Control group mothers could not commit to provide fresh milk daily and were less likely receive antenatal corticosteroids than mothers in the intervention group. In the intervention group, 87.5% (98/112) of mothers were able to provide at least one feed of fresh milk a day. No critical incidents or non-compliance with the protocol were reported. The duration of mechanical ventilation and total parenteral nutrition use were shorter in the intervention group than controls (P < 0.01) but the length of hospital stay was similar (P = 0.57). Although the study might be underpowered, the intervention group had lower unadjusted rates of the composite outcome NEC ≥ stage 2 or mortality (8% vs 20%, P = 0.04), sepsis (22% vs 38%, P = 0.02), retinopathy of prematurity (17% vs 39%, P < 0.01) and bronchopulmonary dysplasia (32% vs 47%, P < 0.01) than the control. These results indicated that feeding fresh mother’s own milk once daily was safe, feasible, and may reduce morbidity.

## Introduction

Necrotizing enterocolitis (NEC) is a severe inflammatory disorder of the intestine that primarily affects very low birth weight (<1500 grams [g]) or very preterm infants (≤32 weeks’ gestation); it is also the leading cause of death in the neonatal intensive care unit (NICU)^1,2^. Currently there is no known effective treatment for NEC. Therefore, the best treatment for NEC is prevention, and mother’s own milk is the best practice for preventing NEC^3^. When mother’s own milk is not available, the options are donor human milk or formula. The use of formula in very low birth weight or preterm infants was reported to increase the risk of developing NEC when compared with donor human milk^4^.

Human milk contains not only nutritional components (such as proteins, amino acids, fats, carbohydrates, vitamins, and minerals), but also a plethora of components with profound bioactivity that are diminished during pasteurisation or freezing of fresh milk. Milk bioactives include both non-cellular (such as cytokines^5^, hormones, growth factors^6^, and oligosaccharides^7^) and cellular components (such as immune cells and stem cells)^8^, many of which exert protective antimicrobial and anti-inflammatory roles in the infant as well as potential developmental effects^9–13^. Recently, Hassiotou (now Kakulas) *et al*. showed that human milk is also a rich source of multilineage stem cells capable of self-renewal and differentiation into cells of all three germ layers^14^. In a mouse study, Kakulas *et al*. demonstrated that similar stem cells exist in mouse milk. Mouse milk stem cells survived in the gastrointestinal tract of the pups after nursing, entered their blood stream, and were incorporated into their major organ systems, where the stem cells were found alive and active (e.g. they contributed to pancreatic insulin production) even after the nursing period was completed^8,15,16^. Although the function and fate of milk stem cells are not yet well understood, it is possible that human milk stem cells benefit the infant through their ability to promote growth, development and regeneration^12^.

In addition, the current evidence strongly suggests that fresh human milk may have a protective effect against both infection and NEC^17^. However, current practices related to the handling and use of human milk in the NICU raise questions about whether we are deriving the maximum benefit from human milk and inversely whether the processing of human milk adversely affects infants. Currently, the standard NICU procedure is to freeze and store milk expressed by mothers of preterm infants in the milk bank. When the infant requires oral feeds, an order is placed with the milk bank and the oldest batch of milk is defrosted, fortifier is added as needed, and then the milk is sent to the NICU for use. The act of freezing human milk (either at −20 °C or −80 °C) along with the passage of time are known to decrease the energy content and reduce the amounts of several useful components of human milk^18,19^, including fat, carbohydrates, secretory immunoglobulin A, lactoperoxidase, lysozyme, antibacterial factors, and antioxidants^18–23^. In addition, the defrosted milk does not contain any live cells, and stem cells in milk have a half-life of 4 hours on average^8,24^.

The current NICU human milk feeding procedure exists as a means of ensuring that infants have consistent access to their mother’s milk even if the mother is not able to spend time in the NICU. The process also allows for strict quality and infection control, as well as computerised inventory and monitoring via electronic health records. However, the process deprives infants of the benefits of the cellular content of human milk, including both the immune cells and stem cells^4^. For the use of fresh human milk in China, there are several barriers to overcome: (1) many hospitals do not allow parents into the NICU during their infant’s stay; (2) mothers may produce less milk than is needed, either initially or throughout the breastfeeding period, so some feeds may need to be supplemented with donor milk or formula; (3) previous reports indicated that fresh human milk may be a source of bacterial and viral contamination, especially by cytomegalovirus (CMV)^25^; however, at the same time numerous more recent studies show that fresh human milk contains a unique microbiome that normally transfers numerous benefits to the developing gastrointestinal tract of the infant^26^.

We performed a multicentre prospective cohort analytic study to evaluate the feasibility and safety of providing very preterm infants born at <30 weeks’ gestation with fresh mother’s own milk within 4 hours of expression. While we acknowledge that the pilot study was not powered to detect a statistically significant difference, our secondary objective was to identify if fresh human milk had the potential to improve infant outcomes, particularly the occurrence of NEC and sepsis. We hypothesized that it is feasible for mothers to provide to their infant at least 1 feed per day of fresh, unprocessed human milk within 4 hours of expression, and that fresh mother’s own milk may decrease the prevalence of NEC and sepsis.

## Materials and Methods

### Study design

The study was a prospective cohort design including infants born at <30 weeks’ gestation at one of four tertiary NICUs in China (Children’s Hospital of Zhengzhou University, Nanjing Maternity and Child Health Hospital, Nanjing Children’s Hospital, and Xiangya Second Hospital). All NICUs were of similar size (≥45 beds) and had donor human milk available for infants born preterm. In the intervention (fresh human milk) group, mothers were asked to provide at least 1 feed of fresh milk within 4 hours of milk expression per day from the time of enrollment until the infants were 32 weeks’ corrected age. The control group included mothers who did not agree to provide fresh milk, but agreed for their infants to receive exclusively human milk (donor or frozen mother’s own milk) and allowed their information to be collected and analyzed.

Our design included several elements to ensure the safety of the mothers and infants. Every infant admitted to the NICU completed the TORCH screen for toxoplasma, rubella, CMV, and herpes simplex^27,28^. If we identified infants as CMV-IgG (+), we completed a human milk CMV-DNA test. We continued to feed the infant fresh human milk if the CMV-DNA level was below 1.0 × 10^4^ copies/ml; however, if the CMV-DNA was above this level, we pasteurized the human milk. For an individual patient, the feeding protocol was stopped if there was an occurrence of NEC or any other medical condition that standard practice required stopping feeds. All cases of mortality, NEC, sepsis, and critical incidence reports (including mix-up of milk destined for individual infants, infection that was related to feeds, missed feeds arising from delays in milk preparation, and any other incident that concerned the clinicians) were critically reviewed by a Data Safety and Monitoring Committee. Stopping criteria were also decided by the Data Safety and Monitoring Committee. The Children’s Hospital of Zhengzhou University was the study coordinating centre, and the study was approved by the research ethics board of each participating hospital and conducted in accordance with their guidelines.

### Study population

The study population included very preterm infants born at <30 weeks’ gestation between January 1, 2016 and December 31, 2017 and admitted to one of the four participating NICUs described above. Infants were eligible if they were born at <30 weeks’ gestation and had never received infant formula. Infants with major congenital anomalies, receiving palliative care, or where illness of the mother or infant prevented the administration of human milk feeds in the first week of the infant’s life were excluded from the study. Eligible mothers were approached at their first visit to the NICU following their infant’s admission and asked to provide informed consent to participate in the study. Only infants whose parents provided informed consent participated in the study. At the time of recruitment, mothers who committed to providing at least 1 feed of fresh human milk a day, 7 days a week, were included in the fresh human milk group, and mothers who allowed their data to be collected and their infants to receive exclusively human milk (donor or frozen mother’s own milk), but were unable to commit to providing at least 1 feed of fresh human milk a day, were included in the control group. If mothers were unable to produce milk for the duration of the study, the infant was fed donor human milk or formula and dropped from the fresh human milk group.

### Feeding intervention

Following standard procedures, all infants in the study were initially provided nutrition intravenously (total parenteral nutrition [TPN]). If the infant was not suffering from any condition that affects the gastrointestinal system function, oral feeds were introduced as early as possible through a nasogastric tube using mother’s own milk or donor human milk. Once the infant was receiving all their nutrition from human milk (‘full enteral feeds’), fortifiers and other nutritional supplements, including probiotics, were added to each feed. Cow’s milk based human milk fortifier was added first, then seven days later vitamins and iron were added to each feed.

All infants were fed following their unit’s feeding procedures and received frozen human milk; however, the fresh human milk group received fresh mother’s own milk instead of frozen milk once a day. Neither the control group nor the fresh human milk group infants were fed formula. For the frozen human milk, all mothers who provided expressed milk followed the standard NICU protocols; nurses picked up the expressed human milk from the mothers, then it was frozen in the milk bank. Each evening the feeds for the following day were ordered, and the oldest batch of human milk was defrosted and prepared. Mothers in the fresh human milk group provided the nurses at least one fresh feed (within 4 hours of milk expression) seven days a week that was fed directly to their infant(s) without freezing or heating until the infants were 32 weeks’ corrected age. Infants were moved from enteral feeds to breastfeeding when they were clinically judged able to do so.

### Data collection

Infant and maternal characteristics, delivery information, and outcomes data were collected on a data collection form and entered by a research assistant into a database for subsequent analysis. Study-specific variables were also collected, including rate of recruitment, rate of consent, rate of retention, compliance with the intervention protocol, the number and volume of fresh milk feeds per infant per day, and the response of each infant following the feed.

The primary outcome of the study was feasibility as measured by the percentage of mothers who provided at least one feed a day of fresh human milk from the time of enrollment until the infant was 32 weeks’ corrected age, the rate of consent to participate in the study, rate of recruitment to the fresh human milk group, and compliance with the intervention protocol. Secondary outcomes included infant growth, mortality, sepsis, NEC, retinopathy of prematurity (ROP), bronchopulmonary dysplasia (BPD), and intraventricular hemorrhage (IVH). A post-hoc analysis included the composite outcome NEC or mortality. Infant growth was measured by the weight z-score and change in weight z-score at 63 days of age^29^. Sepsis was defined on the basis of a positive blood culture and treatment with antibiotics for ≥5 days. NEC was defined as ≥stage 2 according to Bell’s criteria^30^. ROP was defined according to the International Committee for the Classification of Retinopathy of Prematurity^31^. BPD was defined as oxygen need at 36 weeks’ postmenstrual age^32^. IVH grade 3 or 4 and periventricular leukomalacia were defined as described by Papile *et al*.^33^.

### Sample size

The mean number of infants born at <30 weeks’ gestation and admitted to participating NICUs in 2014 was over 500 infants per year. We aimed to include 100 infants in each group and expected that 25% of mothers approached would consent to be in the fresh human milk group. In 2013 the mean annual rate of NEC among participating hospitals was 9.3% in infants born at <29 weeks’ gestation, so we expected to see 9 cases of NEC in the control group. Although the study was not powered to detect a statistically significant difference in outcome rates, the occurrence of 5 or less cases of NEC in the fresh human milk group would be an indication that further study is warranted.

### Statistical analyses

The primary outcomes, secondary outcomes, infant and maternal characteristics were summarized using descriptive methods and compared using the chi-square test for categorical variables and t-test for continuous variables. Multivariable logistic regression analyses were performed to assess the impact of the type of milk on NEC or mortality outcomes. For continuous variables, multiple linear regression analysis was used and adjusted for potential confounders. Crude relative risks (RR) with 95% confidence intervals (95% CIs) were estimated. The level of statistical significance was set at P < 0.05. The SPSS software version 21.0 (SPSS Chicago, Illinois, USA) was used for statistical analysis and data management.

## Results

### Feasibility and safety of the fresh human milk study

**Figure 1:**
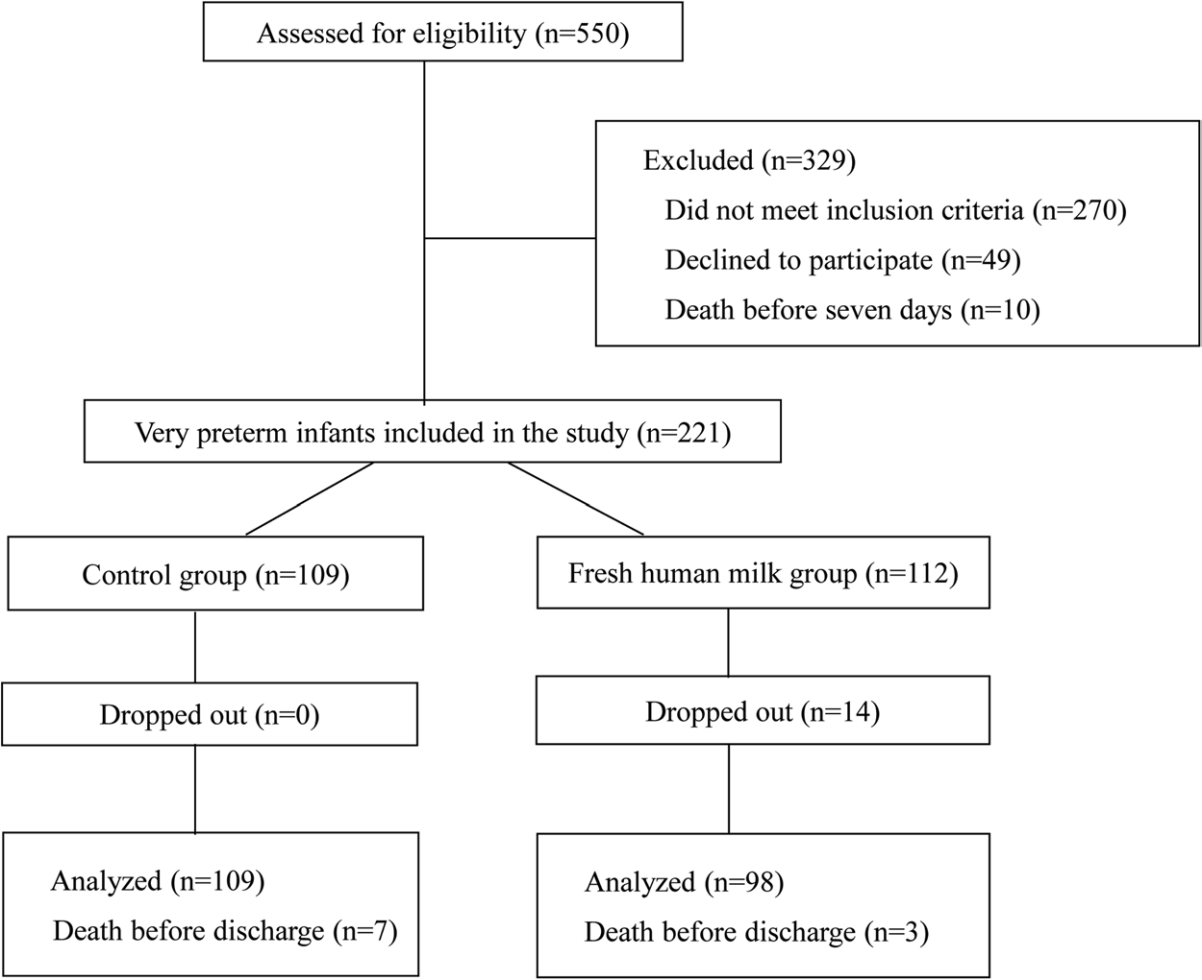
Consort Diagram Flow chart of included infants.

Between January 1, 2016 and December 31, 2017, there were 550 preterm infants born at <30 weeks’ gestation who were assessed for eligibility. Ten of the potentially eligible infants died before 7 days of age, and 270 infants were excluded because they did not meet the inclusion criteria (Fig. 1). Another 49 infants were excluded because their parents declined to participate resulting in a consent rate of 81.9% (221/270). Of the 221 very preterm infants included in this study, we were able to recruit 50.7% (112/220) of consenting mothers to the fresh human milk group and the remaining 109 infants were in the control group. In the fresh human milk group, 14 infants dropped out because their mothers could not produce enough fresh human milk, which means 87.5% (98/112) of mothers in the fresh human milk group were able to provide at least one feed of fresh milk a day. Therefore, we included 98 infants in the fresh human milk group and 109 in the control group in the analyses (Fig. 1).

All NICUs were able to fully comply with the intervention protocol. No critical incidents, including CMV infection or feeding errors, were reported during the study. One mother’s milk contained higher than normal CMV-DNA levels, but the mother was from the control group and her milk was pasteurized. None of the infants in this study were infected with CMV. Furthermore, none of the following incidents were observed in either study group: mix-up of milk destined for individual infants, infection that was related to feeds, missed feeds arising from delays in milk preparation, or any other incident that concerned the clinicians.

### Variation in maternal and infant characteristics

The majority of maternal characteristics were similar between the fresh human milk and control groups, except more mothers in the fresh human milk group had a college diploma or university degree, were treated with antenatal corticosteroids, and received magnesium sulphate during labor (Table 1). Several infant characteristics varied between the fresh human milk and control groups (Table 2). The average Apgar Scores were higher (P < 0.01) and the duration of mechanical ventilation (P < 0.01) and TPN use (P < 0.01) were shorter in the fresh human milk group than in the control group.

**Table 1:** Comparison of maternal characteristics of fresh human milk and control groups.

Maternal Characteristics	Fresh Human Milk N = 98	Control^a^ N = 109	P value
Age of mothers (years), mean ± SD	30.6 ± 5.5	29.8 ± 5.7	0.26
Pregnancy weight (kg), mean ± SD	58.2 ± 7.8	57.2 ± 5.8	0.31
Marital status (married), n (%)	97(99)	107(98)	0.50
Education			<0.01
Elementary school, n (%)	7(7)	7(6)	
High school, n (%)	47(48)	84(77)	
Trade certificate, n (%)	5(5)	10(9)	
Professional registration, n (%)	0	1(1)	
College diploma, n (%)	16(16)	3(3)	
University degree, n (%)	23(23)	4(4)	
Conception by assisted reproductive technology, n (%)	7(7)	17(16)	0.20
Antenatal corticosteroid, n (%)	47(50)	13(12)	<0.01
Gestational diabetes, n (%)	11(11)	4(4)	0.11
Hypertension or pre-eclampsia, n (%)	18(18)	11(10)	0.31
MgSO_4_ during labour, n (%)	25(26)	4(4)	<0.01
Clinical chorioamnionitis, n (%)	8 (8)	7(6)	0.80
Antenatal bleeding, n (%)	10(10)	22(20)	0.14
Postpartum haemorrhage, n (%)	2(2)	8(7)	0.37
Length of ROM, n (%)			0.04
<24 hours	68(69)	91(83)	
24 hours to 1 week	17(17)	10(9)	
>1 week	6(6)	1(1)	
Unknown	7(7)	7(6)	
Labor initiation (spontaneous), n (%)	63(64)	78(72)	0.22
Mode of delivery, n (%)			0.69
Vaginal	64(65)	78(69)	
Assisted vaginal	1(1)	2(2)	
Emergency caesarean	26(27)	22(20)	
Elective Caesarean	7(7)	7(6)	
Presentation (vertex), n (%)	84(86)	99(91)	0.26

^a^Infants in the control group were fed frozen milk.

Abbreviations: N, number in category; n, number in group; SD, standard deviation; ROM, Rupture of membrane.

**Table 2:** Comparison of infant characteristics and treatments in fresh human milk and control groups.

Infant Characteristics	Fresh Human Milk N = 98	Control^a^ N = 109	P value
Age of infant at recruitment (hours), mean ± SD	10.3 ± 6.0	10.8 ± 7.0	0.58
Male sex, n (%)	60(61)	59(54)	0.32
Gestational age at birth (weeks), mean ± SD	28.3 ± 3.6	28.7 ± 1.2	0.20
Apgar Scores			
One minute, mean ± SD	7.3 ± 2.3	5.9 ± 2.3	0.01
Five minutes, mean ± SD	8.2 ± 1.8	7.2 ± 2.1	0.01
Ten minutes, mean ± SD	8.8 ± 1.4	7.9 ± 1.6	0.01
Number of births in pregnancy			
Singleton, n (%)	79(81)	67(61)	0.01
Twins, n (%)	17(17)	39(36)	
Triplets, n (%)	1(1)	3(3)	
Respiratory Support			
CPAP (days), mean ± SD	8.1 ± 7.2	8.9 ± 9.4	0.49
Mechanical Ventilation (days), mean ± SD	2.9 ± 5.3	5.6 ± 6.9	0.01
Oxygen (fraction of inspired oxygen)			
28 days, mean ± SD	24.7 ± 5.0	29.4 ± 6.4	0.01
36 weeks, mean ± SD	27. 2 ± 4.1	28.1 ± 2.7	0.60
Surfactant administration, n (%)	65(66)	74(68)	0.88
Caffeine administration, n (%)	85(87)	86(79)	0.15
TPN(days), mean ± SD	17.3 ± 8.0	21.8 ± 12.7	0.01
Duration of hospitalization (days), mean ± SD	47.0 ± 18.1	48.5 ± 19.6	0.57

^a^Infants in the control group were fed frozen human milk.

Abbreviations: N, number in category; n, number in group; SD, standard deviation CPAP, Continuous Positive Airway Pressure; TPN, Total Parenteral Nutrition.

### Study outcomes

To determine if infant growth was affected by fresh human milk, we assessed infant weight gain and change in weight over the course of the study. The birth weight z-score of the fresh human milk and control groups were similar (P = 0.42), and 9 weeks after birth (63 days) the average weights remained similar for both groups (P = 0.25, Table 3). However, the infants in the fresh milk group gained weight faster than the control group as indicated by the significant difference between the two groups in the change of weight z-score at 63 days of age (P = 0.01, Table 3).

**Table 3:** Comparison of the weight gain of preterm infants in the fresh human milk and control groups.

Weight	Fresh Human Milk (N = 98) mean ± SD	Control^a^ (N = 109) mean ± SD	P value
Birth weight z-score	0.35 ± 0.90	0.25 ± 0.83	0.42
Weight z-score at 7 days of age	−0.38 ± 0.77	−0.11 ± 0.44	0.34
Weight z-score at 14 days of age	−0.35 ± 2.1	−0.71 ± 0.66	0.10
Weight z-score at 21 days of age	−0.70 ± 0.74	−0.64 ± 2.40	0.80
Weight z-score at 28 days of age	−0.92 ± 0.75	−1.08 ± 0.68	0.13
Weight z-score at 35 days of age	−1.19 ± 0.74	−1.28 ± 0.69	0.44
Weight z-score at 42 days of age	−1.51 ± 0.72	−1.58 ± 0.68	0.62
Weight z-score at 49 days of age	−1.70 ± 0.77	−1.74 ± 0.68	0.77
Weight z-score at 56 days of age	−1.93 ± 0.71	−1.97 ± 0.67	0.83
Weight z-score at 63 days of age	−1.91 ± 0.78	−2.17 ± 0.61	0.25
Change in weight z-score^b^	−1.72 ± 0.32	−2.10 ± 0.52	0.01

^a^Infants in the control group were fed frozen human milk.

^b^Z-score at 63 days of age minus z-score at birth.

Abbreviations: N, number in group; SD, standard deviation.

We used linear regression analyses to analyze weight gain velocity and other outcomes after adjusting for confounding factors including gestational age, birth weight, sepsis, and duration of mechanical ventilation. Weight gain velocity was significantly higher in the fresh human milk group than the control group, even after adjusting for confounding factors (Table 4). In addition, the fresh human milk group had a shorter duration of TPN than the control group (P = 0.02; Table 4). A trend was found in favour of the fresh human milk group for duration of hospitalization, but the results were not significant (Table 4).

**Table 4:** Linear regression analysis of the impact of fresh human milk versus control milk on hospital stay, weight gain and total parenteral nutrition.

	Standardized Coefficients	95% CI	
	Beta	Lower Limit	Upper Limit
Duration of hospitalization, days	0.02	−3.97	5.10
Weight gain, g/d	1.90	0.12	3.67
Change in weight z-score^b^	0.27	0.04	0.51
Total parenteral nutrition, days	0.15	0.64	6.02

^a^The fresh human milk group was compared to the control group and the model was adjusted for gestational age, birth weight, sepsis, and duration of mechanical ventilation.

^b^Z-score at 63 days of age minus z-score at birth.

Fresh human milk did not impact the mortality rates, which were similar between the fresh human milk and control groups (Table 5). The incidence of NEC was lower, although not significant, in the fresh human milk group than in the control group. However, the risk of the composite outcome NEC ≥ stage 2 or mortality was significantly lower in the fresh human milk group than in the controls. In addition, significantly fewer infants in the fresh human milk group had sepsis, ROP, or BPD than the control group (Table 5).

**Table 5:** Comparison of the neonatal outcomes of preterm infants fed with and without fresh human milk.

Outcomes	Fresh Human Milk N = 98	Control^a^ N = 109	Relative Risk^b^ (95%CI)	P value
NEC ≥ stage 2 or mortality, n (%)	8(8)	20(18)	0.45(0.21–0.96)	0.04
Mortality, n (%)	3(3)	7(6)	0.48(0.13–1.79)	0.09
NEC ≥ stage 2, n (%)	6(6)	15(14)	0.46(0.19–1.10)	0.11
Sepsis, n (%)	22(22)	41(38)	0.60(0.38–0.93)	0.02
ROP diagnosis, n (%)	17(17)	43(39)	0.44(0.27–0.72)	<0.01
ROP required treatment, n (%)	9(9)	15(14)	0.64(0.30–1.40)	0.29
BPD, n (%)	3(3)	20(18)	0.17(0.05–0.54)	<0.01
IVH ≥ Grade 3, n (%)	6(6)	13(12)	0.51(0.20–1.30)	0.23
PVL, n (%)	5(5)	12(11)	0.46(0.17–1.27)	0.14
RDS, n (%)	85(78)	97(89)	0.98(0.88–1.08)	0.67

^a^Infants in the control group were fed frozen human milk.

^b^The relative risk was calculated by comparing the probability of fresh human milk group to the outcomes of the control group.

Abbreviations: CI, confidence interval; RR, relative risk; NEC, necrotizing enterocolitis; ROP, retinopathy of prematurity; IVH, intraventricular hemorrhage; PVL, periventricular leukomalacia; RDS, respiratory distress syndrome; BPD, bronchopulmonary dysplasia.

## Discussion

Our study indicated that feeding preterm infants (born at <30 weeks’ gestation) fresh human milk, within 4 hours post-expression, is a feasible and safe practice in the NICU. Of the 221included infants, 112 mothers agreed to participate in the fresh human milk intervention. While 14 mothers were unable to produce enough milk and dropped out, the remaining 98 mothers in the fresh human milk group all complied with the intervention protocol. Importantly, we did not have any critical incidents, including CMV infection, throughout the duration of the study. While we acknowledge that our pilot study was not powered to detect a statistically significant difference, our results suggest that only one fresh human milk feed per day has the potential to improve infant outcomes such as sepsis and NEC. Together, these results suggest that the use of fresh human milk in very preterm infants is feasible, safe, and could improve infant outcomes.

Human milk is a fresh, living fluid containing much more than nutrition. It is a cellular, as well as non-cellular, bioactive factory with live stem cells, immune cells, and numerous antioxidants, antibacterial, prebiotic, probiotic, and immune-boosting properties in addition to proteins, essential fats, enzymes, and hormones, all of which are uniquely human^34^. Whenever nursing or immediately expressed milk is unavailable^35^, properly stored human milk continues to provide safe and adequate nutrition, superior to formula feeding^36^, and is the gold standard for infant feeding. However, like any other living tissue or liquid, human milk is sensitive to the effects of temperature^37^, and some nutrients and bioactive properties are adversely affected by storage conditions. For example, pasteurized human milk stored at −20 °C has significantly less fat, lactose, and energy content than fresh human milk^38^. Furthermore, freezing human milk at −80 °C significantly decreases the energy content from both fat and carbohydrates lower than the levels observed in human milk stored at −20 °C^19^. Last, freezing depletes the live immune and stem cells in human milk, which are known to provide protective and developmental benefits to the infant^11,12^.

One potential problem with fresh human milk is the risk of CMV infection, which is of particular concern in the case of prematurity^39^; however, the topic is controversial and guidelines vary. Often, pasteurization of mother’s own milk is recommended for preterm infants because of the potential risk of CMV contamination. In our pilot study, we did not detect any CMV infection in the infants in the control or fresh human milk groups suggesting that it is safe to feed preterm infants fresh human milk.

Given previous reports that associated human milk with a decrease in NEC^40,41^, the NEC outcomes in our study were interesting. Keeping in mind the limitations in our study design, fresh human milk was protective against NEC when compared to the control, which is in agreement with a previous study showing that an early and rapid increase in enteral feeding with human milk was associated with a decreased risk of NEC^41^. Furthermore, a Belgian randomised controlled trial (RCT) pointed out that with fresh milk rather than pasteurised milk there was a lower frequency of NEC that required surgery^40^.

The potential of fresh human milk to reduce the incidence of BPD is also intriguing. Previous studies showed that oxidative stress is a major causative factor of BPD^42^, and fresh human milk has antioxidant properties^43^ that are altered by pasteurisation^44^. For example, fresh milk contains vitamins C and E and enzymes; including superoxide dismutase, catalase, and glutathione peroxidase; that are known to protect against the potentially-harmful effects of oxidative stress^45^. Our study supports further investigating the impact of fresh human milk on the incidence of BPD in a future RCT.

There are several limitations with our study. First, we observed differences in the baseline characteristics between the fresh human milk and control groups that we could not control for because our pilot study was under-powered. The educational level of mothers and the incidence of antenatal corticosteroid and magnesium sulphate use were higher in the fresh human milk group than the control group. It is possible that the better antenatal care and understanding of the benefits of fresh human milk by the mothers in the fresh human milk group impacted the infant outcomes in this group. Furthermore, the infants in the fresh human milk group had higher Apgar scores, were more likely to be singletons, spent fewer days on mechanical ventilation, and fewer days on TPN than the control group, all of which could have led to better outcomes for the fresh human milk group. Last, the definition of NEC is still heterogeneous and depends on the clinical judgement of the physician.

Despite the limitations, our study indicates that it is feasible and safe to feed preterm infants <30 weeks’ gestation fresh human milk. In China, there are no recent recommendations regarding the use of mother’s own fresh milk in NICUs. Practices differ widely among NICUs, but a high number of centres use fresh human milk to feed very preterm infants in recent years. The American Academy of Pediatrics recommends that all preterm infants are routinely fed fresh mother’s own milk^13^. To further evaluate the benefits of fresh human milk, an RCT is warranted.

The lessons learned from our study will provide important insights for our future RCT. For example, in China parents are not allowed in the NICU with their infants, which can make the logistics of providing fresh milk challenging. In our study, mothers consented to provide at least one feed of fresh human milk a day, and we allowed mothers into the NICU to pump to ensure human milk was delivered expediently. Future RCTs should also ensure mothers have a place in the NICU to pump human milk to enable compliance with the fresh human milk requirements. Furthermore, our estimate that 25% of mothers would consent to be in the fresh human milk group was low. We found that 81.9% (221/270) consented to participate in the study and 50.7% (112/220) of consenting mothers agreed to be in the fresh human milk group, which is the number that should be used for sample size calculations in the future. In a future multicenter randomized trial we will need to use multi-level models adjusting for potential confounders to analyze the data.

## Conclusion

In summary, our multicentre prospective cohort study found that feeding preterm infants <30 weeks’ gestation fresh mother’s own milk once a day was safe and feasible in the NICU. Furthermore, our results suggested a potential reduced risk of NEC, sepsis, ROP, and BPD for these infants. Going forward, a large randomized multicentre study is needed to analyze the impact of fresh human milk on the outcomes of infants born preterm. If fresh human milk is shown to improve preterm infant outcomes, it will prompt new milk preparation and feeding guidelines for these vulnerable infants.

## Data Availability

The datasets generated during and/or analysed during the current study are available from the corresponding author on reasonable request.
